# Disrupted‐in‐schizophrenia 1 overexpression disrupts hippocampal coding and oscillatory synchronization

**DOI:** 10.1002/hipo.23076

**Published:** 2019-02-05

**Authors:** Karola Kaefer, Hugo Malagon‐Vina, Desiree D. Dickerson, Joseph O'Neill, Svenja V. Trossbach, Carsten Korth, Jozsef Csicsvari

**Affiliations:** ^1^ Present address: Institute of Science and Technology Austria (IST Austria) Am Campus 1, Klosterneuburg Austria; ^2^ School of Psychology Cardiff University 70 Park Place, Cardiff United Kingdom; ^3^ Department Neuropathology, Medical Faculty Heinrich Heine University Düsseldorf Moorenstrasse 5, Düsseldorf Germany

**Keywords:** DISC1, electrophysiology, hippocampus, oscillations, place cells, protein pathology

## Abstract

Aberrant proteostasis of protein aggregation may lead to behavior disorders including chronic mental illnesses (CMI). Furthermore, the neuronal activity alterations that underlie CMI are not well understood. We recorded the local field potential and single‐unit activity of the hippocampal CA1 region in vivo in rats transgenically overexpressing the *Disrupted‐in‐Schizophrenia 1* (*DISC1*) gene (tgDISC1), modeling sporadic CMI. These tgDISC1 rats have previously been shown to exhibit DISC1 protein aggregation, disturbances in the dopaminergic system and attention‐related deficits. Recordings were performed during exploration of familiar and novel open field environments and during sleep, allowing investigation of neuronal abnormalities in unconstrained behavior. Compared to controls, tgDISC1 place cells exhibited smaller place fields and decreased speed‐modulation of their firing rates, demonstrating altered spatial coding and deficits in encoding location‐independent sensory inputs. Oscillation analyses showed that tgDISC1 pyramidal neurons had higher theta phase locking strength during novelty, limiting their phase coding ability. However, their mean theta phases were more variable at the population level, reducing oscillatory network synchronization. Finally, tgDISC1 pyramidal neurons showed a lack of novelty‐induced shift in their preferred theta and gamma firing phases, indicating deficits in coding of novel environments with oscillatory firing. By combining single cell and neuronal population analyses, we link DISC1 protein pathology with abnormal hippocampal neural coding and network synchrony, and thereby gain a more comprehensive understanding of CMI mechanisms.

## 1 INTRODUCTION

Chronic mental illnesses (CMI), such as schizophrenia and recurrent affective disorders, are highly heritable and have been associated with a large number of genetic loci (Schizophrenia Working Group of the Psychiatric Genomics Consortium, 2014). However, there is agreement that not all causes of CMI are heritable, indicating that the pathophysiology underlying CMI is complex and also involves nongenetic factors (Pardiñas et al., 2018). Albeit still a niche in psychiatry research, there is increasing evidence that aberrant protein homeostasis and resulting aggregation may be a nongenetic cause for developing CMI (Bradshaw & Korth, 2018). Such proteinopathies can thereby lead to CMI when extracellular stressors cause misassembly of initially healthy proteins that have inherently unstructured domains, making this disease mechanism not rely on genetic variation.

Disrupted‐in‐schizophrenia 1 (DISC1) is such an unstructured domain protein and has been associated with CMI in several populations (Chubb, Bradshaw, Soares, Porteous, & Millar, 2007; Millar et al., 2000). Although there is still disagreement in the field whether mutated *DISC1* is a genetic cause of CMI (Porteous et al., 2014; Sullivan, 2013), there is considerable evidence that DISC1 protein pathology may play a role in schizophrenia and other CMI. Indeed, it has been demonstrated that DISC1 possesses a propensity for aggregation and misassembly (Atkin, Brandon, & Kittler, 2012; Trossbach et al., 2016; Wen et al., 2014). Furthermore, a subgroup of *postmortem* brains of patients diagnosed with affective disorders or schizophrenia expressed significant fractions of DISC1 protein aggregates (Leliveld et al., 2008). Based on these findings, a rat model has been developed that transgenically overexpresses nonmutant, full‐length DISC1 in the central nervous system (tgDISC1 rat), leading to aggregates of DISC1 protein, a dopamine system disturbance in brain areas including the hippocampus and dopamine‐ and attention‐related behavioral deficits (Trossbach et al., 2016; Wang et al., 2017). The tgDISC1 rat examines the effect of DISC1 protein pathology, unlike previous models that tested the effect of DISC1 mutation (Jaaro‐Peled, 2009).

Cognitive deficits are prominent symptoms of CMI (Millan et al., 2012). The hippocampus is likely to play a key role in these deficits since this brain area is involved in various cognitive processes and morphological and functional alterations have been found in the hippocampus of CMI patients (Harrison, 2004; Heckers et al., 1998; MacQueen et al., 2003). The cognitive functions of the hippocampus are supported by concerted neuronal activity that often follows rhythmic activation patterns and that is fundamental for establishing precise temporal coordination (Buzsáki & Draguhn, 2004). Theta (6–10 Hz) and gamma band (40–100 Hz) oscillations are found in the hippocampus (Buzsáki, 2002; Buzsáki & Draguhn, 2004) and aid spatial and mnemonic coding (Howard et al., 2003; Huxter, Burgess, & O'Keefe, 2003; Huxter, Senior, Allen, & Csicsvari, 2008; O'Keefe & Recce, 1993; Raghavachari et al., 2001). Sharp wave ripple (SWR) oscillations (150–250 Hz) occur during sleep and waking immobility (Buzsaki, Horvath, Urioste, Hetke, & Wise, 1992; Csicsvari, Hirase, Czurkó, Mamiya, & Buzsáki, 1999a; Ylinen et al., 1995) and have been linked to memory consolidation (Dupret, O'Neill, Pleydell‐Bouverie, & Csicsvari, 2010; Ego‐Stengel & Wilson, 2010). A growing body of evidence suggests that abnormal oscillatory network dynamics might underlie some CMI and cause the impaired cognitive functions present in schizophrenia patients (Buzsáki & Watson, 2012; Jones, 2010; Phillips et al., 2012; Suh, Foster, Davoudi, Wilson, & Tonegawa, 2013; Uhlhaas & Singer, 2010).

In rodents, the most widely demonstrated role for the hippocampus is its involvement in spatial cognition. Hippocampal principal cells encode spatial information and jointly provide a neural representation of the external environment (O'Keefe & Nadel, 1978). The spatial firing of these place cells, however, is not only influenced by the location of the environment, but also by nonspatial information such as cognitive demands and episodic‐like experiences (Allen, Rawlins, Bannerman, & Csicsvari, 2012; Eichenbaum, Dudchenko, Wood, Shapiro, & Tanila, 1999; Leutgeb et al., 2005).

We hypothesized that DISC1 misassembly and the resulting disturbance of DISC1‐dependent signaling pathways in the hippocampus might lead to alterations in hippocampal coding on a single cell and network level, which in turn might underlie some of the behavioral phenotypes seen in the tgDISC1 rat (Trossbach et al., 2016; Wang et al., 2017). Thereby, place coding could be affected, but also coding of place‐independent variables and oscillatory dynamics. We performed in vivo recordings of multiple single‐units and local field potentials (LFPs) in the dorsal CA1 hippocampus in tgDISC1 and control rats during exploration of familiar and novel open field environments and subsequent sleep. This study shows that tgDISC1 CA1 place cells exhibit altered spatial coding, while also displaying deficits in coding of location‐independent features. Furthermore, analyses of the neuronal population activity relative to oscillations revealed disturbances in network flexibility and synchrony in tgDISC1 rats, leading to deficits in adapting to novel environments and its consolidation. Finally, tgDISC1 pyramidal cells displayed reduced firing during SWRs.

We therefore demonstrate that aggregation of a nonmutant protein and resulting downstream effects can lead to a wide range of impairments in hippocampal neural circuit function, which may ultimately underlie behavioral deficits.

## 2 MATERIALS AND METHODS

### 2.1 Subjects and tetrodes implantation

All procedures involving experimental animals were carried out in accordance with Austrian animal law (Austrian federal Law for experiments with live animals) under a project license approved by the Austrian Federal Science Ministry. TgDISC1 and wildtype littermate control animals were generated by breeding two DISC1 heterozygous Sprague Dawley rats. For all animals used always one male tgDISC1 and one male littermate control rat originated from the same litter of a single breeding pair (Wang et al., 2017). Genotypes were identified by genetic screening as described in Trossbach et al. (2016).

Animals were implanted with microdrives housing 16 independently movable tetrodes targeting the right dorsal CA1 region of the hippocampus. Each tetrode was fabricated out of four 12 um tungsten wires (California Fine Wire Company, Grover Beach, CA) that were twisted and then heated to bind into a single bundle. The tips of the tetrodes were gold‐plated to reduce the impedance to 300–450 kΩ. During surgery, the animal was under deep anesthesia using isoflurane (0.5–3%), oxygen (1–2 L/min), and an initial injection of buprenorphine (0.1 mg/kg). A rectangular craniotomy was drilled at −3.4 to −5 mm AP and − 1.6 to −3.6 mm ML relative to bregma. Five to six anchoring screws were fixed onto the skull and two ground screws were positioned above the cerebellum. After removal of the dura, the tetrodes were initially implanted at a depth of 1–1.5 mm relative to the brain surface. Finally, the microdrive was anchored to the skull and screws with dental cement. Two hours before the end of surgery the analgesic Metacam (5 mg/kg) was given. After a one‐week recovery period, tetrodes were gradually moved into the dorsal CA1 cell layer.

### 2.2 Behavior

Animals were housed individually in a separate room under a 12 hr light/12 hr dark cycle with ad libitum access to water and were maintained in a food‐deprived state between 85 and 90% of their postoperative weight. Two to three days before the start of recording, animals were familiarized with a circular open‐field environment (diameter = 80 cm). On each recording day, the animal underwent a behavioral protocol in the following order: 10 min resting in a bin located next to the open‐field environment, exploration of the familiar open‐field environment (20 min), sleep/rest in the familiar open‐field environment (20 min), exploration of a novel open‐field environment (20 min), sleep/rest in the novel open‐field environment (20 min). Whilst the familiar environment was kept constant, the novel environment differed on every recording day. The novel open‐field arenas differed in their floor and wall linings, and shapes. The recordings for the familiar and novel conditions were performed in the same recording room. During all open‐field explorations, food pellets (20 mg) were scattered on the floor to encourage foraging and therefore good coverage of the environment. Only recording days that upon visual inspection had extensive exploratory coverage were used for analysis.

### 2.3 Data acquisition

A headstage (2 × 32 channels, Axona Ltd, St. Albans, Hertfordshire, UK) was used to pre‐amplify the extracellular electric signals from the tetrodes. Wide‐band (0.4 Hz–5 kHz) recordings were taken and the amplified LFP and multiple‐unit activity were continuously digitized at 24 kHz using a 64‐channel data acquisition system (Axona Ltd). Two red LED bundles mounted on the preamplifier head‐stage were used to track the location of the animal.

### 2.4 Spike sorting and unit classification

Clustering of spikes and unit isolation procedures were described previously (Csicsvari, Hirase, Czurko, & Buzsáki, 1998). Briefly, the raw data was resampled to 20 kHz and action potentials were detected from the digitally high‐pass‐filtered (0.8–5 Hz) signal. The power was then computed in a sliding window (12.8 ms) and action potentials with a power of >5 *SD*s from the baseline mean were extracted. Using principal component analysis the action potential features were extracted. The action potentials were then grouped into multiple putative units based on their spike features using an automatic clustering software (http://klustakwik.sourceforge.net; Harris, Henze, Csicsvari, Hirase, & Buzsáki, 2000). The generated clusters were then manually refined using a graphical cluster‐cutting program. Only units with clear refractory periods in their autocorrelation, well‐defined cluster boundaries and stability over time were used for further analysis. Cluster stability was verified by plotting spike features over time and an isolation distance (based on Mahalanobis distance) was calculated to ensure that spike clusters did not overlap (Harris et al., 2000). Excitatory CA1 pyramidal cells and inhibitory interneurons were discriminated using their auto‐correlograms, firing rates and waveforms. In our analysis, we included 864 cells for tgDISC1 (721 pyramidal cells and 143 interneurons) and 688 for control (541 pyramidals and 147 interneurons) animals. For analyses involving place cells, we only included pyramidal cells with spatial firing fields that met a coherence (>0.5) and sparsity (<0.3) criteria (Muller & Kubie, 1989; Skaggs, McNaughton, Wilson, & Barnes, 1996). For tgDISC1 animals: 373 (familiar environment) and 343 (novel environment), control animals: 228 (familiar environment) and 235 (novel environment) place cells were identified.

### 2.5 Behavior analysis

To determine the movement speed we first calculated the distance travelled between two consecutive 25.6 ms time windows and then computed the average from 10 such consecutive distances, excluding zeros. Multiplying these distances by the sampling rate resulted in an average speed for every 256 ms time window. These values were then averaged to obtain the average speed within a behavioral session. To calculate immobility the distance travelled within 1 s time windows was computed. The percentage of immobility was obtained by dividing the number of distances less than 2 cm by the total number of time windows. To calculate open‐field coverage the tracking data was binned into 5 cm bins and each tracking coordinate was allocated to the respective bin of an empty matrix. To differentiate between empty bins within and outside the open‐field arena, an empty bin was declared a nonvisited area within the arena if less than 5 other empty bins surrounded it. The percent open‐field not covered by the animal was calculated as the fraction of nonvisited bins over all the bins within the open‐field.

### 2.6 Rate remapping

Rate remapping scores were calculated over all pyramidal neurons using the absolute difference between their firing rates from two conditions divided by their sum:

$$\text{Remapping score}=\frac{\left|{\mathrm{FR}}_{C1}-{\mathrm{FR}}_{C2}\right|}{{\mathrm{FR}}_{C1}+{\mathrm{FR}}_{C2}}$$

where *FR*_*C*1_ and *FR*_*C*2_ is the average firing rate during Conditions 1 and 2, respectively. This score was calculated for the following: familiar versus novel, first versus second half of familiar and first versus second half of novel environment.

### 2.7 Place map analysis

Firing rate maps were computed by dividing the open‐field environments into 70 × 70 bins. A cell's firing rate was calculated for every spatial bin by counting the number of occurred spikes and dividing them by the animal's time spent in that bin. Raw rate maps were speed‐filtered (5 cm/s) and smoothed using a two‐dimensional Gaussian kernel.

The spatial tuning of cells was calculated with Skaggs spatial information measure (Skaggs, McNaughton, Gothard, & Markus, 1993). The place field size is equal to the ratio between the number of bins spanned by the place field of the cell (all bins with a firing rate >0.1 times the maximum firing rate), divided by the number of total bins covered by the animal during the exploration of the environment.

The index of dispersion is the ratio between the variance and the mean of the number of spikes occurred in each passing of the animal through the place field of the neuron. For the speed versus firing rate analysis, the instantaneous firing rate counts (IFRCs) of place cells and the corresponding speed of the animal were measured during 500 ms windows during exploration.

### 2.8 Bayesian decoding

We used Bayesian place prediction (Zhang, Ginzburg, McNaughton, & Sejnowski, 1998) to investigate if, together with increased spatial information, place cells of tgDISC1 animals allow better reconstruction of the animal's position during exploration. We established population vectors in 250 ms windows (125 ms overlap, each containing at least 1 spike) while the animal was exploring the open environment. Rate maps built over the whole task provide a firing probability for each spatial bin. The formula below gives the probability that a given population vector represents a given place:

$$P\left(x|n\right)=P\left(n|x\right)P\left(x\right)/P\left(n\right)$$

*P*(*x*) represents the probability that the animal is at a given location considering the exploration session was set to a uniform distribution to not bias our analysis by any place preference of the animal (Zhang et al., 1998). *P*(*n*|*x*) represents the conditional probability that a given spike count occurs at a location. This was estimated using the firing rates of the place‐rate maps, assuming that the number of spikes follow a Poisson distribution. *P*(*n*), the normalizing constant, was used to ensure that *P*(*x*|*n*) summed up to 1. The location with the maximum probability was selected as the reconstructed position. Error measurements represented the absolute distance between the middle of the reconstructed bin to the real position of the animal.

### 2.9 Noise correlation analysis

For the noise correlation, the exploratory IFRCs of cells were calculated separately for different time windows occurring at different spatial bins (environment was divided into 20 × 20 equal sized bins). Then, the correlation of IFRCs between cell pairs was calculated separately for each spatial bin and averaged across all bins to yield the noise correlation.

### 2.10 Speed‐compensated noise correlation

For the speed‐compensated noise correlation, the exploratory IFRCs of cells was calculated separately for different time windows occurring at different spatial bins (environment was divided into 20 × 20 equal sized bins). The speed of the animal was additionally measured for each time window in which the IFRCs were taken. The cofiring was then calculated as the speed‐compensated partial correlation of the IFRCs of a cell pair.

### 2.11 Oscillation analyses

Theta periods in exploration and rapid eye movement (REM) sleep were detected based on the theta (6–10 Hz) and delta (2–4 Hz) power ratio as previously described (Csicsvari, Hirase, Czurkó, Mamiya, & Buzsáki, 1999b). The theta/delta ratio was measured in 1600 ms segments (800 ms steps in between measurement windows), using Thomson's multi‐taper method. To detect theta oscillations, the LFP was filtered in the 5–28 Hz band. Gamma periods were extracted by digitally filtering the LFP in the 30–80 Hz band. Then for each electrode, the root mean square error was calculated within a 25 ms window. The gamma periods were defined as those with a power of 2 *SD* above the mean. SWRs were detected by band‐pass filtering (150–250 Hz) the LFP and subtracting a reference signal (from a channel without SWRs placed above the CA1 pyramidal layer) to eliminate common noise‐like muscle artifacts. The power (root mean square) of the filtered signal was calculated for each electrode and summed across all electrodes that were in the CA1 pyramidal layer. The threshold for SWRs detection was set to 7 *SD* above the background mean and was set in the first available sleep session.

For phase locking analysis we detected the oscillation phases at which spikes occurred. From the distribution of these phases a mean vector was calculated for each neuron. The mean vector has a length and an angle, providing information about the neurons' phase locking strength (variance of firing phases) and preferred firing phase, respectively. Each arrow in Figures 3a—d, 4a—d, and 5c,d corresponds to the mean vector of one neuron. To calculate the mean phase locking strength for the recorded neurons, the mean of all mean vector lengths was computed (Figures 3e,i, 4e,i, and 5e). To estimate the concentration of the preferred firing phases (Figures 3f,j and 4f,j) we computed the vector length (*r*) of all preferred phases of significantly locked neurons (*p* < 0.05). This was done by taking the mean over the rectangular coordinates of all angles: $X=\frac{\sum_{i=1}^{n}\cos a_{i}}{n}$ and $Y=\frac{\sum_{i=1}^{n}\sin a_{i}}{n}$, where ‘*n*’ is the total amount of neurons and ‘*a*’ is the angle component of that neuron. Then *r*, also known as concentration value, was calculated as $r=\sqrt{X^{2}+Y^{2}}$ (Zar, 2007). To compare the concentration values between the group distributions, we converted r into an angular variance *S*^2^ = 1 − *r* and then compared the variances with a variance‐ratio test.

The confidence intervals of the angular variance, for an alpha of 0.05, were calculated as follows:

$$\text{Upper}\;\mathrm{CI}=1-\frac{\left(n-1\right)S^{2}}{\chi_{0.975,\left(n-1\right)}^{2}}$$

$$\text{Lower}\;\mathrm{CI}=1-\frac{\left(n-1\right)S^{2}}{\chi_{0.025,\left(n-1\right)}^{2}}$$

For gamma‐theta locking analysis the peak of every gamma cycle was detected. Then, if the gamma cycle occurred during theta oscillations, the theta angle at which the gamma cycle peaked was extracted. From all these extracted theta angles the preferred theta angle was then computed.

### 2.12 Power spectral density

Power spectral density was calculated using the Welch's method. Thereby a discrete Fourier transform of the windowed (Hanning windows, 500 ms, 50% overlap) LFP (0–500 Hz) was performed. In order to compare the power between sessions, the power was normalized over the sum. Power calculations were done for frequency bins corresponding to 1 Hz each.

### 2.13 Reactivation

We assessed reactivation in sleep/rest SWRs by testing whether the tendency of cell pairs to fire together during SWRs was similar to that in previous waking exploration. To measure the strength of their joint firing tendency (cofiring), we first established for each cell its IFRCs in 100‐ms windows during theta (exploratory session) and SWR (sleep/rest session) periods. The correlation coefficient between the IFRCs for each pair was then calculated separately for exploration and sleep/rest. The similarity of cell pairs' cofiring tendency across exploration and sleep/rest sessions was then assessed by calculating the correlation coefficient between exploration and sleep/rest period cofiring.

### 2.14 Statistical analysis

All analyses were performed in Matlab, Octave, and C. All statistics were performed with the corresponding parametric tests, except those where the data did not pass a test for normality (Kolmogorov–Smirnov test). In those cases, the nonparametric equivalent was used. Post hoc correction for multiple comparisons was applied when necessary (Bonferroni‐Holm correction). Statistical tests and *P* values are stated in the respective figure legends and/or in the results section. *P* values of *p ≤ 0.05, **p ≤ 0.01, ***p ≤ 0.001 were used as significance levels.

## 3 RESULTS

### 3.1 Altered spatial coding of tgDISC1 place cells during open‐field exploration

**Figure 1:**
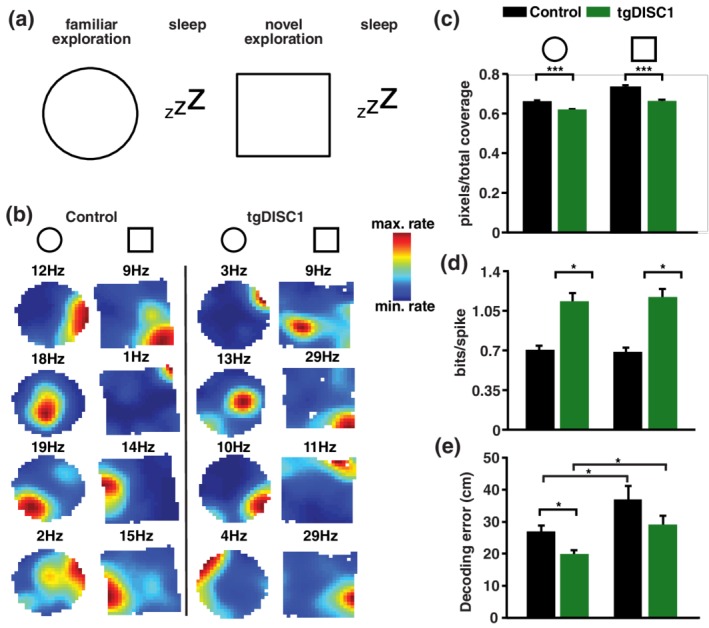
Altered spatial coding in tgDISC1 animals. (a) Animals explored a familiar and a novel open‐field environment, each followed by a sleep session. The novel environment varied for every recording day. (b) Place fields of example place cells for control (left panel) and tgDISC1 animals (right panel), during familiar (circle) and novel (square) environment exploration. Each row shows a different neuron. The maximum firing rate of every neuron during respective environment exploration is indicated above. Each map is scaled to the maximum firing rate of the neuron. Note the smaller place fields of tgDISC1 neurons. (c) Place cells recorded in tgDISC1 have smaller place field sizes compared to control place cells, in both the familiar and novel environment (Wilcoxon rank‐sum test, Bonferroni‐Holm correction; familiar: *p* = 5.70e − 26, *N*_control_ = 228 cells, *N*_tgDISC1_ = 373 cells, novel: *p* = 2.27e − 16, *N*_control_ = 235 cells, *N*_tgDISC1_ = 343 cells; d) Higher spatial information content in pyramidal cells of tgDISC1 compared to control animals during familiar and novel environment explorations (Wilcoxon rank‐sum test, Bonferroni‐Holm correction; familiar: *p* = 0.024, novel: *p* = 0.02, *N*_control_ = 541 cells, *N*_tgDISC1_ = 721 cells). (e) Bayesian decoding error of spatial position using place cell firing. The red line indicates the median and the lower and upper limits of the box indicate the 25th and 75th percentiles, respectively. The lower and upper error bars extend to the smallest and largest observation, respectively. Decoding in the familiar environment was significantly more accurate for tgDISC1 animals compared to controls (Wilcoxon rank‐sum test, Bonferroni‐Holm correction; familiar: *p* = 0.014) and decreased in the novel environment for both groups (Wilcoxon signed‐rank test, Bonferroni‐Holm correction; control: *p* = 0.032, tgDISC1: *p* = 0.024). Unless otherwise stated data are represented as mean and standard error of mean (*SEM*) [Color figure can be viewed at wileyonlinelibrary.com]

Four tgDISC1 and four control rats were implanted with 16 independently movable tetrodes targeting the CA1 region of the dorsal hippocampus. Multiple units and LFP recordings were performed while animals explored a familiar and a novel open field environment and rested before and after exploration in sleep/rest sessions (Figure 1a). Analysis of behavior on the open‐field showed decreased movement speed and increased immobility in tgDISC1 compared to controls (Wilcoxon rank‐sum test, Bonferroni‐Holm correction; speed—familiar: *p* = 0.0002, novel: *p* = 0.01; immobility—familiar: *p* = 0.012, novel: *p* = 0.022; Supporting Information Figure S1a,b). However, the percent of environment not covered was less than 6 % and did not differ between the groups (Wilcoxon rank‐sum test, familiar: *p* = 0.127, novel: *p* = 0.226; Supporting Information Figure S1,c). We recorded the activity of 864 neurons (721 pyramidal cells and 143 interneurons) in tgDISC1 and 688 neurons (541 pyramidal cells and 147 interneurons) in control animals. From these pyramidal neurons, 373 and 343 place cells were identified in tgDISC1 animals during exploration of the familiar and novel environment respectively, while in control animals 228 place cells were recorded in the familiar and 235 in the novel environment. To test if novelty is reflected in neuronal activity, we performed rate remapping analyses between pyramidal neuron firing related to familiar and novel environment exploration, and also, as a control, between the first and second halves of both explorations. For tgDISC1 and controls, switching from a familiar to novel environment resulted in significantly stronger rate remapping than when comparing rates from the first and second half of any environment exploration (Two‐sample Kolmogorov–Smirnov test; control: *P*_1st‐2nd‐familiar_ = 3.55e − 16, *P*_1st‐2nd‐novel_ = 1e − 15, tgDISC1: *P*_1st‐2nd‐familiar_ = 5.45e − 30, *P*_1st‐2nd‐novel_ = 5.47e − 61; Supporting Information Figure S2). Analyzing spatial properties of cells showed that place fields of place cells were significantly smaller in tgDISC1 animals compared to controls in both environments (Wilcoxon Rank‐Sum test, Bonferroni‐Holm correction; familiar: *p* = 5.70e − 26, novel: *p* = 2.27e − 16; Figure 1b,c). In line with the place field size results, the activity of pyramidal cells held higher spatial information content in tgDISC1 than in control animals (Wilcoxon Rank‐Sum test, Bonferroni‐Holm correction; familiar: *p* = 0.024, novel: *p* = 0.021; Figure 1d). To assess the effect of smaller place fields and higher spatial information content on the ability to code for space, we performed a Bayesian decoding analysis (see Section 2). We found that in the familiar environment decoding the spatial position of an animal based on the firing of recorded place cells was significantly better in tgDISC1 than controls (Wilcoxon rank‐sum test, Bonferroni‐Holm correction; familiar: *p* = 0.014; Figure 1e). For the novel environment we could see a trend in this direction (Wilcoxon rank‐sum test, Bonferroni‐Holm correction; novel: *p* = 0.0771). Prediction became less accurate in the novel environment for both groups (Wilcoxon signed‐rank test, Bonferroni‐Holm correction; control: *p* = 0.032, tgDISC1: *p* = 0.024), indicated by the increase in decoding error. Together these findings show that tgDISC1 animals exhibited altered spatial coding and that smaller place fields enable better prediction of spatial position based on neural firing.

### 3.2 Impaired location‐independent coding of tgDISC1 place cells

**Figure 2:**
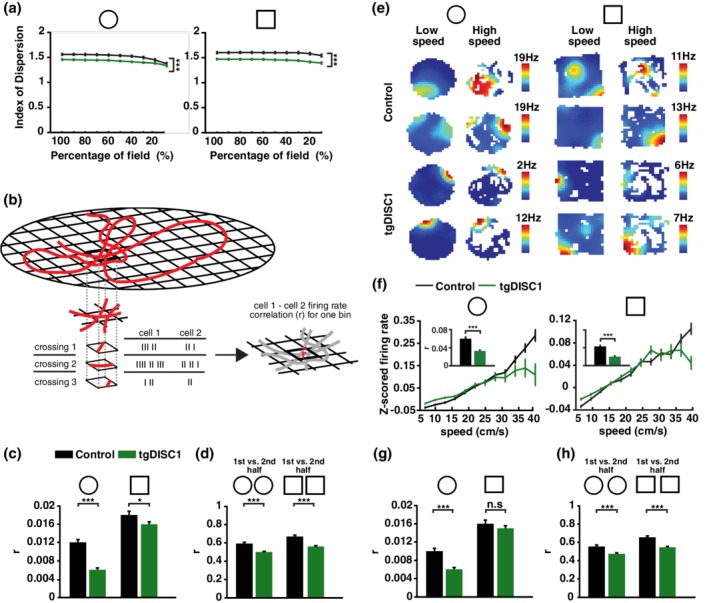
Impaired location‐independent coding in tgDISC1 animals. (a) Index of dispersion for different percentages of the place cell firing fields for control (black) and tgDISC1 (green) animals during familiar (circle) and novel (square) environment explorations. The *x* axis indicates the calculation for the different place field size fractions. Note that tgDISC1 place cells have a lower index of dispersion in both the familiar and novel environments (two‐way ANOVA; familiar: *F*(1,9) = 135.24, *p* = 6.31e − 31, *N*_control_ = 228 cells, *N*_tgDISC1_ = 373 cells, novel: *F*(1,9) = 298.53, *p* = 3.02e − 65, *N*_control_ = 235 cells, *N*_tgDISC1_ = 343 cells). (b) Schema of noise correlation calculations. The red line indicates a path taken by the animal in a given environment (grid). (c) Noise correlation of place cell pairs is higher in control than tgDISC1 animals for both familiar and novel environment explorations (One‐way ANOVA of Pearson correlation coefficients, Bonferroni‐Holm correction; familiar: *F*(1,9652) = 59.626, *p* < 0.0001, *N*_control_ = 2,439 cell pairs, *N*_tgDISC1_ = 7,215 cell pairs, novel: *F*(1,9,650) = 4.093, *p* = 0.043, *N*_control_ = 2,865 cell pairs, *N*_tgDISC1_ = 6,787 cell pairs). (d) The correlation between the noise correlations of the first and second half of environment exploration was lower for tgDISC1 place cell pairs than cell pairs of control animals, for both the familiar and novel environment conditions (*Z*‐test of Fisher *z*‐transformed Pearson correlation coefficients, Bonferroni‐Holm correction; familiar: *p* < 0.001, novel: *p* < 0.001. Number of cell pairs as in c). (e) Firing rate maps of place cells plotted separately for periods when the rat was moving at low (5–11 cm/s) and high (11–17 cm/s) speeds. White pixels indicate spatial bins that were not visited at that particular speed range. Place cells recorded in control animals (first two rows) exhibited a stronger speed modulation of their firing rate in both the familiar (left) and novel (right) environments compared to place cells recorded in tgDISC1 animals (last two rows). Scale bar shows the maximum firing rate of each neuron. (f) *Z*‐scored instantaneous firing rate of place cells correlated with the running speed of the animal. Small inset bar‐plots: speed versus *z*‐scored firing rate correlations averaged over all place cells. In both, the familiar (circle) and novel (square) environments, tgDISC1 place cell firing rates were less speed‐modulated than the firing rates of control place cells (One‐way ANOVA of Pearson correlation coefficients, Bonferroni‐Holm correction; familiar: *F*(1,592) = 19.918, *p* < 0.0001, *N*_control_ = 227 cells, *N*_tgDISC1_ = 367 cells, novel: *F*(1,625) = 18.11, *p* < 0.0001, *N*_control_ = 255 cells, *N*_tgDISC1_ = 372 cells). (g) Speed‐compensated noise correlation of place cell pairs for the familiar (circle) and novel (square) environments (One‐way ANOVA of Pearson correlation coefficients; familiar: *F*(1,9,652) = 32.446, *p* < 0.0001, novel: *F*(1,9650) = 1.288, *p* = 0.256. (h) The correlation between the speed‐compensated noise correlations of the first and second half of environment exploration is higher for control (black) than tgDISC1 (green) place cell pairs for both the familiar (circle) and novel (square) environment conditions (*Z*‐test of Fisher *z*‐transformed Pearson correlation coefficients, Bonferroni‐Holm correction; familiar: *p* < 0.001, novel: *p* < 0.001). Data are presented as mean and *SEM* [Color figure can be viewed at wileyonlinelibrary.com]

Given that the spatial information content of pyramidal cells was almost twice as high in tgDISC1 than in control animals, the relatively small differences in place field size may not fully explain the differences in spatial information. We tested the variability of place cell firing rates at different passes through the same place fields. We reasoned that reduced spatial information content in control pyramidal cells might arise due to firing rate variability that codes for location‐independent information. To quantify the variability of spatial firing, we measured the index of dispersion (i.e., the ratio of the variance and mean of firing rates of a place cell at different passes through the same place field), which was significantly higher for control than for tgDISC1 animals (Two‐way ANOVA; familiar: *F*(1,9) = 135.24, *p* = 6.31e − 31, novel: *F*(1,9) = 298.53, *p* = 3.02e − 65; Figure 2a). This suggests that in control animals place cells have a better ability to encode information beyond the location of the animal by modulating their firing rates (Fenton & Muller, 1998; Leutgeb et al., 2005). To test whether these rate variations were random or generated by cells consistently modulating their rates at different locations, we performed noise correlation analysis. The noise correlation analysis used here measures the strength of firing coupling between pairs of cells, independently of their spatial selectivity (Figure 2b, see Section 2). The noise correlation of place cells was significantly higher for control animals in both environment explorations (One‐way ANOVA of Pearson correlation coefficients; familiar: *F*(1,9,652) = 59.626, *p* < 0.0001, novel: *F*(1,9,650) = 4.093, *p* = 0.043; Figure 2c). In addition, we also tested whether the noise correlation structure of place cells remained similar while animals explored an environment. When the correlation structure of place cell pairs was compared in the first and second halves of exploration, they were stronger correlated in control than in tgDISC1 animals, for both familiar and novel environment (*Z*‐test of Fisher *z*‐transformed Pearson correlation coefficients, Bonferroni‐Holm correction; familiar: *p* < 0.001, novel: *p* < 0.001; Figure 2d). These results demonstrate that control animals exhibit stronger cell assembly‐specific modulation of their firing rates, independent of place.

Such consistent place‐independent modulation of place cell activity may be related to location‐independent sensory experience of the animal such as the speed‐related firing rate increase of place cells (McNaughton, Barnes, & O'Keefe, 1983; Wiener, Paul, & Eichenbaum, 1989). Indeed, place cells of tgDISC1 animals exhibited weaker speed modulation of their firing rate compared to control place cells (One‐way ANOVA of Pearson correlation coefficients, Bonferroni‐Holm correction; familiar: *F*(1,592) = 19.918, *p* < 0.0001, novel: *F*(1,625) = 18.11, *p* < 0.0001; Figure 2e,f). This directly demonstrates a deficit in tgDISC1 animals to encode information not directly linked to location.

Given that tgDISC1 animals exhibited weaker speed‐modulated firing raised the possibility that the observed differences in noise correlations were solely due to the differences in speed correlations. To test this, we calculated the noise correlation values while compensating for speed. The speed‐compensated noise correlation values of tgDISC1 and control animals were now only significantly different in the familiar environment, suggesting that differences in coding of nonspatial variables beyond speed were only present with increasing experience of an environment (One‐way ANOVA of Pearson correlation coefficients; familiar: *F*(1,9,652) = 32.446, *p* < 0.0001, novel: *F*(1,9,650) = 1.288, *p* = 0.256; Figure 2g).

However, the configuration of noise correlation patterns compensated by the speed was still more stable in control animals in both environments (*Z*‐test of Fisher *z*‐transformed Pearson correlation coefficients, Bonferroni‐Holm correction; familiar: *p* < 0.001, novel: *p* < 0.001; Figure 2h), suggesting that location‐ and speed‐independent firing patterns of place cells are less stable in tgDISC1 animals.

### 3.3 TgDISC1 neurons show impaired theta and gamma oscillation‐related network interactions

**Figure 3:**
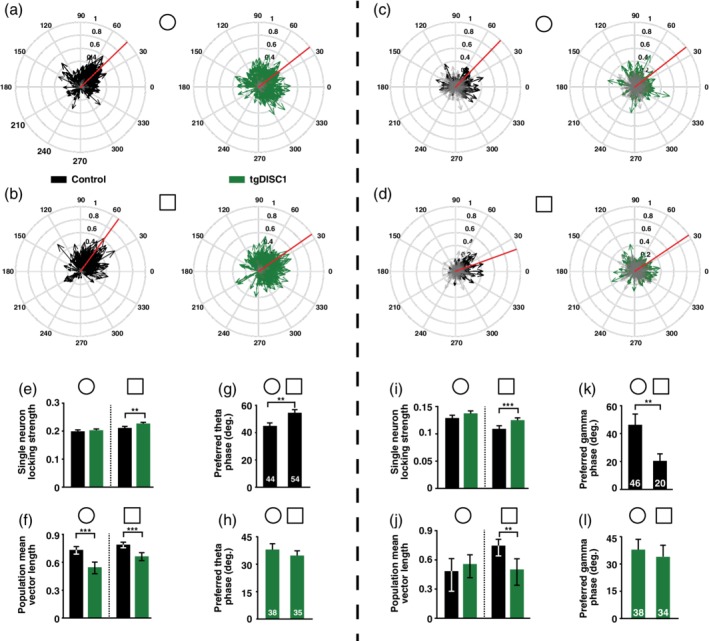
TgDISC1 pyramidal neurons show impaired theta and gamma oscillation‐related network interactions. (a) Circular phase plots of the coupling of pyramidal neurons to theta oscillations during exploration of familiar environment (circle) for control and tgDISC1 animals. Each arrow represents the preferred firing phase and locking strength of a single pyramidal cell. Grey arrows represent not significantly coupled pyramidal cells (*p* > 0.05). The red line shows the mean of all preferred theta firing phases. (b) Same as (a), but during exploration of the novel environment (square). (c) Same as (a), but for coupling to gamma oscillations. (d) Same as (b), but for coupling to gamma oscillations. (e) Comparison of mean theta phase locking strength of the pyramidal neurons shown in (a) and (b) between control and tgDISC1 animals during familiar (circle) and novel (square) environment explorations. Stars indicate a significantly stronger average phase locking of tgDISC1 pyramidal neurons during the novel environment (Wilcoxon rank‐sum test, Bonferroni‐Holm correction; *p* = 0.0044). (f) Comparison of the concentration of preferred theta phases of significantly locked pyramidal cells shown in (a) and (b) for control and tgDISC1 animals during familiar and novel environment exploration. Note that the concentration of preferred theta phases is significantly larger in control than tgDISC1 animals in both environments (Variance‐ratio test, Bonferroni‐Holm correction; familiar: *p* = 4.47e − 7, novel: *p* = 2.48e − 6). Error bars denote confidence intervals for an alpha of 0.05. (g) Comparison of the preferred theta firing phase of pyramidal neurons in control animals exploring the familiar and novel environment. Note the significant phase shift (Watson‐Williams test; *p* = 0.0023). (h) Same as (g), but for pyramidal neurons of tgDISC1 animals. (i) Same as (e), but for gamma oscillations (Wilcoxon rank‐sum test, Bonferroni‐Holm correction; novel: *p* = 5.66e − 04). (j) Same as (f), but for gamma oscillations. Note that the concentration of preferred gamma phases is significantly larger in control than tgDISC1 animals in the novel environment (Variance‐ratio test, Bonferroni‐Holm correction; *p* = 0.0021). (k) Same as (g), but for gamma oscillations. Note the significant phase shift (Watson‐Williams test; *p* = 0.0073). (l) Same as (h), but for gamma oscillations. (theta analyses, familiar: *N*_control_ = 346 cells, *N*_tgDISC1_ = 416 cells, novel: *N*_control_ = 379 cells, *N*_tgDISC1_ = 479 cells; gamma analyses, familiar: *N*_control_ = 81 cells, *N*_tgDISC1_ = 118 cells, novel: *N*_control_ = 76 cells, *N*_tgDISC1_ = 112 cells). Unless otherwise stated data are presented as mean and *SEM* [Color figure can be viewed at wileyonlinelibrary.com]

Neurons in tgDISC1 animals exhibited deficits in simultaneously encoding location‐dependent and location‐independent sensory information. This brings the question whether further alterations in the hippocampal neuronal network activity of tgDISC1 rats can be identified, indicating coding deficits at a wider network scale (Huxter et al., 2003, 2008; O'Keefe & Recce, 1993; Raghavachari et al., 2001). We detected the theta and gamma phases at which the spikes of pyramidal cells occurred. The coupling of the pyramidal cell population to theta and gamma oscillations is shown by the distributions of mean vectors (see Section 2), each representing one single neuron (Figure 3a–d). A mean vector indicates both the preferred phase (angle) and phase locking strength (length) of one neuron. To measure the mean phase locking strength the mean vector lengths of single neurons were averaged. In the novel, but not in the familiar environment, the locking strength during theta and gamma oscillations was on average stronger for tgDISC1 pyramidal neurons than pyramidal neurons recorded in control animals (Wilcoxon rank‐sum test; theta: *p* = 0.0044, gamma: *p* = 5.66e − 4; Figure 3e,i). This shows that during novelty in tgDISC1 animals, spikes from one pyramidal cell occur all at similar phases making tgDISC1 pyramidal neurons fire more rigidly near their preferred theta and gamma phases compared to control pyramidal neurons.

We then measured the concentration of the preferred phases of recorded pyramidal neurons that were significantly locked (*p* < 0.05) by computing the mean vector over their distribution of preferred theta and gamma phases. The length of this vector (*r*) is considered a measurement of the concentration of the distribution (Zar, 2007) and was significantly lower for tgDISC1 than control pyramidal cells (statistics performed on angular variance, *S* ^2^ = 1 − *r*, variance‐ratio test; theta (familiar): *p* = 4.47e − 7, theta (novel): *p* = 2.48e − 6, gamma (novel): *p* = 0.0021; Figure 3f,j). This indicates that the oscillation phases at which different pyramidal cells fired preferentially were more variable between tgDISC1 pyramidal neurons than between those of controls.

**Figure 4:**
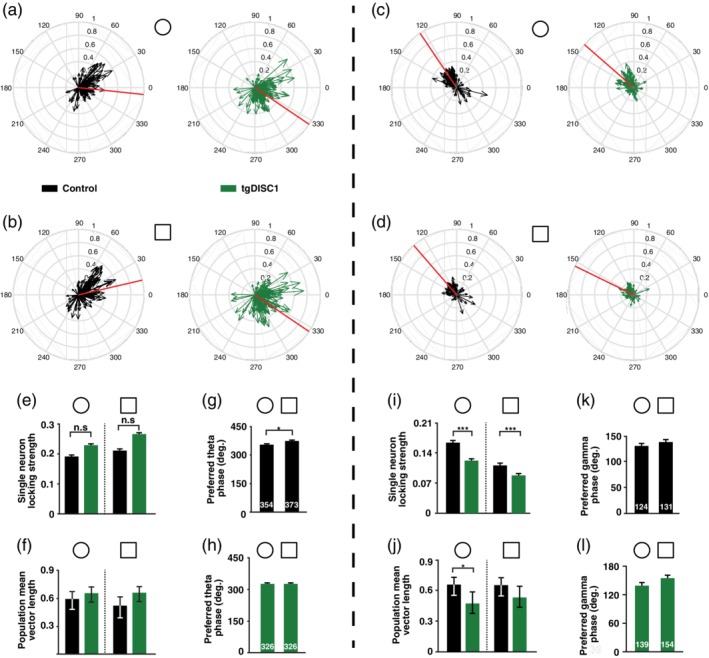
Differences in interneuron responses during theta and gamma oscillations between control and tgDISC1 animals. (a) Circular phase plots of interneuron coupling to theta oscillations during exploration of familiar environment (circle) for control and tgDISC1 animals. Each arrow represents the preferred firing phase and locking strength of a single interneuron. Grey arrows represent not significantly coupled interneurons (*p* > 0.05). The red line shows the mean of all preferred theta firing phases. (b) Same as (a), but during exploration of the novel environment (square). (c) Same as (a), but for coupling to gamma oscillations. (d) Same as (b), but for coupling to gamma oscillations. (e) Comparison of mean theta phase locking strength of interneurons shown in (a) and (b) between control and tgDISC1 animals during familiar (circle) and novel (square) environment explorations. (f) Comparison of the concentration of preferred theta phases of significantly locked interneurons shown in (a) and (b) for control and tgDISC1 animals during familiar and novel environment exploration. Error bars denote confidence intervals for an alpha of 0.05. (g) Comparison of the preferred theta firing phase of interneurons in control animals exploring the familiar and novel environment. Note that interneurons also present a significant shift in their locking phase from familiar to novel environment exploration (Watson‐Williams test; *p* = 0.0113). (h) Same as (g), but for interneurons of tgDISC1 animals. (i) Same as (e), but for gamma oscillations. There are significant differences between the mean locking strength of control and tgDISC1 interneurons (Wilcoxon rank‐sum test, Bonferroni‐Holm correction; familiar: *p* = 2.75e − 5, novel: *p* = 5.05e − 4). (j) Same as (f), but for gamma oscillations. Note that the concentration of preferred phases is significantly different between controls and tgDISC1 for the familiar environment (Variance‐ratio test, Bonferroni‐Holm correction; *p* = 0.019). (k) Same as (g), but for gamma oscillations. (l) Same as (h), but for gamma oscillations. (theta analyses, familiar: *N*_control_ = 145 cells, *N*_tgDISC1_ = 143 cells, novel: *N*_control_ = 145 cells, *N*_tgDISC1_ = 142 cells; gamma analyses, familiar: *N*_control_ = 127 cells, *N*_tgDISC1_ = 119 cells, novel: *N*_control_ = 128 cells, *N*_tgDISC1_ = 108 cells). Unless otherwise stated data are presented as mean and *SEM* [Color figure can be viewed at wileyonlinelibrary.com]

It has previously been shown that when animals experience a novel environment, neurons present a novelty‐induced shift in their preferred theta phase of firing (Lever et al., 2010). Whereas pyramidal cells of control animals showed the expected shift, this was absent in tgDISC1 pyramidal cells (Watson‐Williams test; controls: *p* = 0.0023; Figure 3g,h). In addition, pyramidal neurons of control animals also exhibited a novelty‐induced shift in their preferred gamma firing phase, which was absent in tgDISC1 neurons (Watson‐Williams test; *p* = 0.0073; Figure 3k,l). Same analyses applied to interneurons **(**Figure 4 **)** showed a lack of novelty‐induced shift of the preferred theta firing phase in tgDISC1 animals (Watson‐Williams test; *p* = 0.0113; Figure 4g,h). In addition, interneurons in tgDISC1 animals exhibited weaker phase locking to gamma oscillations in both familiar and novel environments (Wilcoxon Rank‐Sum test; familiar: *p* = 2.75e − 5, novel: *p* = 5.05e − 4; Figure 4i).

The phase locking properties of cells to field oscillations may not be directly related to the field power of an oscillatory band. Therefore, we also compared differences in theta or gamma LFP power. Theta power during familiar and novel exploration was not different between the groups. However, there was a significant increase in gamma power in tgDISC1 animals compared to controls when exploring the familiar environment (Wilcoxon rank‐sum test, *p* = 0.0052; Supporting Information Figure S3). In addition, we also compared gamma phase locking to theta oscillations, by comparing the theta phase distribution of detected gamma oscillatory waves, but did not find any differences between groups (Supporting Information Figure S4).

Overall these results advocate for the hypothesis that tgDISC1 animals exhibit not only an increased network rigidity in temporal coding when novel contexts are presented, but also suggests a network synchronization deficit.

### 3.4 Impaired tgDISC1 neuronal responses during sleep/rest sessions

**Figure 5:**
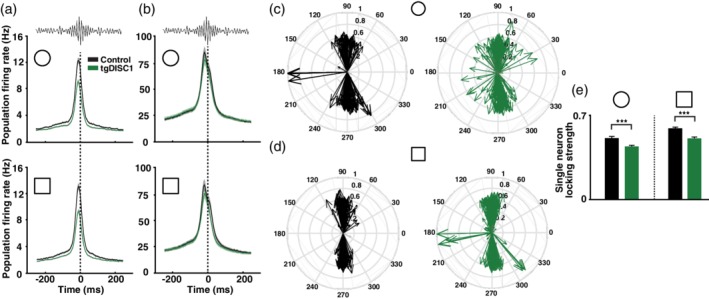
Neuronal activity during SWRs and REM theta oscillations (a) Population firing rate of pyramidal neurons during SWRs for both control and tgDISC1 animals during familiar (circle) and novel (square) environment exploration. Solid lines indicate the mean firing rate and shaded areas the confidence intervals (Wilcoxon rank‐sum test; familiar: *p* = 2.74e − 11, novel: *p* = 1.13e − 12, *N*_control_ = 541 cells, *N*_tgDISC1_ = 721 cells). Dotted lines mark the center of the SWR. (b) Same as (a), but for interneurons (Wilcoxon rank‐sum test; familiar: *p* = 0.44, novel: *p* = 0.498, *N*_control_ = 147 cells, *N*_tgDISC1_ = 143 cells). Data are presented as mean and confidence intervals. (c) Circular phase plots of the coupling of pyramidal neurons to theta oscillations during REM sleep after exploration of the familiar environment (circle) for controls (black arrows, left plot) and tgDISC1s (green arrows, right plot). Each arrow represents the preferred firing phase and locking strength of a single pyramidal cell. Grey arrows represent not significantly coupled pyramidal cells (*p* > 0.05). (d) Same as (c), but during REM theta occurring in sleep after the exploration of the novel environment (square). (e) Comparison of mean REM theta phase locking strength of pyramidal neurons shown in (c) and (d) between control (black) and tgDISC1 (green) animals during sleep following familiar (circle) and novel (square) environment exploration. Stars indicate a significantly stronger average phase locking of control pyramidal neurons to REM theta occurring in sleep following both familiar and novel environment exploration (Wilcoxon rank‐sum test, Bonferroni‐Holm correction; familiar: *p* = 2.59e − 6, novel: *p* = 1.55e − 7). Data are presented as mean and *SEM* [Color figure can be viewed at wileyonlinelibrary.com]

The differences seen in neuronal coding during exploration, lead us to investigate whether these differences also extended to immobility periods in sleep/rest sessions that followed the explorations. We analyzed the neuronal firing responses of pyramidal cells during SWRs, a measure independent of confounding factors such as sleep quality (O'Neill, Senior, & Csicsvari, 2006). We found that pyramidal cells of tgDISC1 animals had a lower firing rate during SWRs occurring in the sleep/rest session after both the familiar and novel environment exploration (Wilcoxon Rank‐Sum test; familiar: *p* = 2.74e − 11, novel: *p* = 1.13e − 12; Figure 5a). Repeating this analysis for interneurons showed no differences (Wilcoxon rank‐sum test; familiar: *p* = 0.44, novel: *p* = 0.498; Figure 5b).

Given that sleep and rest periods are associated with the consolidation of memories and that the hippocampus replays the waking activity patterns of neurons during these times, we also tested whether reactivation was altered during the sleep/rest SWRs. To evaluate the reactivation of place cells, we compared correlated patterns of place cells during awake and in SWRs during sleep/rest periods and found that tgDISC1 animals showed signfiicanlty higher reactivation strength in sleep after both environments compared to controls (*Z*‐test of Fisher *z*‐transformed Pearson correlation coefficients, Bonferroni‐Holm correction; familiar: *p* < 0.001, novel: *p* < 0.001; Supporting Information Figure S5).

Whilst the function of REM sleep is still unclear there is evidence that hippocampal REM theta oscillations are linked to memory consolidation (Boyce, Glasgow, Williams, & Adamantidis, 2016). Based on preferred REM theta locking phase, we could distinguish between two pyramidal neuron populations in both controls and tgDISC1s (Figure 5c,d). These two populations have been described previously and result from CA1 deep and superficial layers exhibiting distinct phase locking preferences during REM theta (Mizuseki, Diba, Pastalkova, & Buzsáki, 2011). We found that REM theta locking strength of pyramidal neurons was higher in control animals during sleep following both familiar and novel environment exploration (Wilcoxon rank‐sum test; familiar: *p* = 2.59e − 6, novel: *p* = 1.55e − 7; Figure 5e). Overall, these results suggest that neuronal network deficits of tgDISC1 rats were not only present during cognition, but also extend to rest and sleep periods.

## 4 DISCUSSION

Our results demonstrate that the aggregation of DISC1 protein, a model of sporadic CMI, leads to alterations in hippocampal CA1 activity patterns at both individual cell and population levels. Place cells of tgDISC1 animals exhibited smaller place fields and less variability in their spatial firing, accompanied by other deficits in location‐independent coding. Analysis of cell firing in relation to awake oscillations demonstrated that tgDISC1 deficits extend to the network level, and included impairments in phase coding ability, specifically during novel environment exploration. TgDISC1 pyramidal cells varied more in their preferred theta firing phases and showed a reduction in their firing rate during SWRs, both indicating a network synchronization deficit.

What mechanism might underlie smaller place fields and impaired encoding of location‐independent information seen in tgDISC1 animals? Computational models elucidating the mechanisms behind rate remapping found that rate modulation and firing field size of place cells depend on the excitatory interaction between the spatial inputs of the medial entorhinal cortex and the nonspatial inputs of the lateral entorhinal cortex (Rennó‐Costa, Lisman, & Verschure, 2010). Alternatively, larger CA1 place fields are found in HCN1 and CA1‐specific NMDAR1 knock‐out (KO) mice (Hussaini, Kempadoo, Thuault, Siegelbaum, & Kandel, 2011; McHugh, Blum, Tsien, Tonegawa, & Wilson, 1996). Thus, smaller place fields and reduced place cell firing variability, resulting from DISC1 protein aggregation and downstream effects, might be due to a disturbance in entorhinal cortex‐hippocampal input balance or a reduction in the overall excitability of these cells.

In our animal model the overexpression of nonmutant DISC1 and resulting DISC1 protein aggregation lead to behavioral and molecular phenotypes indicating a dopamine system disturbance: amphetamine supersensitivity, a stark increase in high‐affinity D2 receptors, an increase in dopamine inflow in the dorsal striatum, decreased dopamine concentrations in brain areas including the hippocampus (Trossbach et al., 2016), and decreased numbers of dopaminergic neurons in the substantia nigra and projections (Hamburg et al., 2016). Importantly, CMI are linked to disturbances in dopaminergic transmission (Creese, Burt, & Snyder, 1976; Davis, Kahn, Ko, & Davidson, 1991; Harrison & Weinberger, 2005). In agreement with the dopamine phenotype, tgDISC1 animals exhibit impairments in a striatum‐dependent motor task and object‐recognition requiring spatial attention (Trossbach et al., 2016; Wang et al., 2017) where latter deficit could be compensated by intranasal dopamine administration.

One cognitive dysfunction commonly associated with CMI patients is a deficit in attention (Cornblatt & Keilp, 1994; Smith & Cornblatt, 2005). Switching of spatial attention underlies variation in place cell firing in rats: animals focusing on both intra‐ and extra‐maze cues of an open‐field environment showed more variable place cell firing than animals that learned to focus only on extra‐maze cues by ignoring intra‐maze changes of wall enclosure color patterns (Fenton et al., 2010). Dopamine contributes to spatial attention: place cells in D1R‐KO mice do not exhibit place field remapping upon changes to distal cues, making them only respondent to proximal cues (Tran et al., 2008). Therefore attention deficits resulting from disturbances in the dopamine system may lead to the decreased variability of place cell firing observed in tgDISC1 animals and consequently contribute to a more rigid spatial coding.

In addition to spatial attention, novelty has been shown to lower the threshold for the induction of LTP, a facilitation that is dependent on dopamine receptor activation (Li, Cullen, Anwyl, & Rowan, 2003). Therefore, our finding that tgDISC1 pyramidal cells exhibited stronger phase locking to both theta and gamma oscillations, exclusively for the novel environment, indicates rigidity in network activity in conditions where plasticity is usually increased. Since we could only report power differences in gamma oscillations during familiar exploration, the mentioned phase locking results cannot be linked to differences in theta or gamma power. Failure to adapt to a novel environment, reflected by decreased rate remapping of tgDISC1 pyramidal neurons between the first and second halves of novel environment exploration, further indicates novelty‐related neural coding rigidity in tgDISC1 rats.

In our work we show that tgDISC1 place cells have smaller place fields, hence exhibiting altered spatial coding properties. Place cell goal‐coding deficits were reported in a mouse model where *reduced* DISC1 protein expression leads to a dopamine system disturbance, providing further evidence for dopaminergic involvement (Hayashi, Sawa, & Hikida, 2016). Interestingly, impaired space‐related place cell dynamics have also been observed in CMI models not based on DISC1 manipulations (Wolff & Bilkey, 2015; Zaremba et al., 2017) and amyloidosis‐based Alzheimer's disease models (Mably, Gereke, Jones, & Colgin, 2016; Zhao, Fowler, Chiang, Ji, & Jankowsky, 2014). Our study therefore supports previous CMI research involving place cell coding deficits. However, the reduction in the variability of in‐field firing and in the cell assembly‐specific firing rate modulation, together with decreased speed‐firing rate correlation of place cells in tgDISC1 animals, highlight the importance of investigating location‐independent coding in animal models of mental illness.

Schizophrenia has been theorized as a disorder of coordinated network activity because patients commonly show impairments in neural oscillations (Uhlhaas & Singer, 2010). Indeed, network synchrony disturbances have also been detected in rodent schizophrenia models that showed disrupted theta oscillation synchrony between the hippocampus and prefrontal cortex (Dickerson, Wolff, & Bilkey, 2010; Sigurdsson, Stark, Karayiorgou, Gogos, & Gordon, 2010) or disrupted cortical spindle and hippocampal SWR coordination in sleep (Phillips et al., 2012). Furthermore, mice expressing truncated DISC1 display theta and low gamma synchrony impairments in the prefrontal cortex (Sauer, Strüber, & Bartos, 2015). Here, pyramidal neurons of tgDISC1 animals had a broader preferred firing phase distribution for both gamma and theta oscillations, demonstrating a coordination deficit between neurons and, as suggested by previous work, pointing to a synchronization disturbance within the hippocampus and with other brain areas (von Stein & Sarnthein, 2000).

Sleep disturbances, specifically circadian rhythm disruptions, reduced cortical sleep spindles and impairments in sleep‐dependent memory consolidation are commonly reported for schizophrenia patients (Demanuele et al., 2017; Wamsley et al., 2012; Wulff, Gatti, Wettstein, & Foster, 2010). Studies investigating the sleep architecture of rodent schizophrenia models have found disrupted temporal coupling of hippocampal SWRs to prelimbic spindles and a twofold increase in SWRs in the maternal‐immune activation and calcineurin KO model, respectively (Phillips et al., 2012; Suh et al., 2013). We showed that pyramidal neurons in tgDISC1 animals exhibited a firing rate reduction during hippocampal SWRs and weaker phase locking to REM theta, indicating impaired sleep network activity patterns and information processing also in tgDISC1 animals. Furthermore, tgDISC1 animals showed increased reactivation in sleep compared to controls. This was unexpected, since it has previously been shown that stimulating dopaminergic hippocampal projections in prior waking periods improves reactivation (McNamara, Tejero‐Cantero, Trouche, Campo‐Urriza, & Dupret, 2014). Our rat model, however, showed lower DA levels, indicating that increased tgDISC1 reactivation in tgDISC1 likely results from different molecular mechanisms.

Schizophrenia and recurrent affective disorders have also been linked to cholinergic system disturbances (Cannon et al., 2006; Dilsaver, 1986; Gibbons, Scarr, McLean, Sundram, & Dean, 2009; Raedler, Bymaster, Tandon, Copolov, & Dean, 2006) and the levels of this neurotransmitter are low in tgDISC1 rat brain areas including the hippocampus (Wang et al., 2017). Hippocampal pyramidal neurons show a novelty‐induced shift in their preferred theta firing phase (Lever et al., 2010), which is dependent on acetylcholine (Douchamps, Jeewajee, Blundell, Burgess, & Lever, 2013) and favors encoding of new information (Hasselmo, Bodelón, & Wyble, 2002). We showed that this type of shift does not only occur for pyramidal neurons during theta oscillations, but also during gamma oscillations. In addition, interneurons also showed a shift in their preferred theta firing phase. Such theta and gamma oscillation‐associated shifts were not seen in tgDISC1 animals, demonstrating a deficit in the encoding of new information and a disturbance of network synchronicity processes underlying novelty.

Altogether, like in mutation‐based CMI animal models, nonmutant DISC1 aggregation in our model led to alterations in place cell coding, oscillation synchrony and sleep processes, underscoring the impact of aberrant protein homoeostasis and highlighting protein pathology as an unrecognized possible mechanism underlying CMI. These multifaceted hippocampal circuit alterations are subtle and may therefore not affect performance at simpler behaviors, but brain network synchronization and coding abnormalities are expected to underlie deficits in increasingly demanding cognitive tasks requiring focused attention (Wang et al., 2017). Our tgDISC1 animals exhibited normal spatial behaviors and did not show deficits in simple spatial tasks (Trossbach et al., 2016). Interestingly, the lack of overt behavioral deficits, despite the presence of clear neuronal abnormalities, can also be seen in unaffected siblings of schizophrenia patients. These often exhibit phenotypes typical for schizophrenia patients, for example, a reduction in task‐evoked gamma power (Lisman, 2016), MRI brain volume abnormalities (Ho, 2007) and increased amplitude and reduced synchronization of spontaneous macroscopic neuronal activity (Liu et al., 2016).

We here found neural network alterations in hippocampal CA1 that may provide the link between protein aggregation and the behavioral deficits described for tgDISC1 rats. We also show that animal models of CMI require in‐depth electrophysiological investigations that can reveal disease markers underlying complex cognitive deficits. Such electrophysiological alterations can uncover neural network disturbances that, if recognized and mitigated, may improve CMI diagnosis and reduce the risk of disease onset.

## CONFLICT OF INTEREST

The authors declare no conflict of interest.

## Supporting information


**Supporting Information Figure S1 Behavior on the open‐field. (A)** Movement speed (cm/sec) of control and tgDISC1 in the familiar and novel environments. In both environments tgDISC1 animals exhibited significantly lower speed (Wilcoxon Rank‐Sum test, Bonferroni‐Holm correction; familiar: *p* = 0.0002, novel: *p* = 0.01). **(B)** The percentage of immobility periods during familiar and novel environment exposure. Note that only periods where the animal moved more than 5 cm/s were included in the waking place field and oscillatory analyses. In both environments tgDISC1 rats spent significantly more time in immobility (Wilcoxon Rank‐Sum test, Bonferroni‐Holm correction; familiar: *p* = 0.012, novel: *p* = 0.022). **(C)** Percentage of the area of familiar and novel environments that were not visited by the animal. There were no differences in environment coverage between control and tgDISC1 animals (Wilcoxon Rank‐Sum test, familiar: *p* = 0.127, novel: *p* = 0.226).
**Supporting Information Figure S2: Novel environment exploration induces rate remapping.** Cumulative probability distributions of the rate remapping scores for three different comparisons: familiar vs. novel (black), 1st vs. 2nd half of the familiar environment (blue) and 1st vs. 2nd half of the novel environment (red), for both control (left panel) and tgDISC1 (right panel) animals. Comparisons between the distributions are summarized in each panel (Two‐sample Kolmogorov–Smirnov test, P‐values are notated in the figure).
**Supporting Information Figure S3: TgDISC1 animals showed increased gamma power during familiar environment exploration as compared to controls.** Normalized logarithmic power of the local field potential (0–500 Hz) for control (black) and tgDISC1 (green) animals during the exploration of familiar (top) and novel (bottom) environments is shown. Each line represents one recording session. The boxplots inside the panels denote the power distributions of the 1 Hz frequency bins comprising the theta (6–10 Hz) and gamma (30–80 Hz) bands from all sessions. Stars indicate a significant difference between controls and tgDISC1 animals in the gamma band during the exploration of the familiar environment (Wilcoxon Rank‐Sum test, Bonferroni‐Holm correction; *p* = 0.0052).
**Supporting Information Figure S4: Gamma oscillations lock differentially to theta in familiar and novel environments. (A)** Normalized histogram of the preferred theta phase (blue line represents two theta cycles) of gamma wave peaks, for control (black) and tgDISC1 (green) animals during familiar exploration. **(B)** Phase plots of the preferred theta locking phase of detected gamma wave peaks in familiar (black arrow) and novel (red arrow) environment, for controls (top) and tgDISC1 animals (bottom). Both groups of animals showed a significant shift of preferred theta locking phase of gamma waves (Watson‐Williams test; controls: familiar (240°), novel (220°), *p* < 1e‐30, tgDISC1: familiar (239°), novel (224°), *p* < 1e‐30). **(C)** Same as **A,** but during novel environment exploration.
**Supporting Information Figure S5: Reactivation during rest period SWRs**. TgDISC1 animals showed significantly higher reactivation strength in sleep after both environments compared to controls (Z‐test of Fisher z‐transformed Pearson correlation coefficients, Bonferroni‐Holm correction; familiar: *p* < 0.001, N_control_ = 1,331 cell pairs, N_tgDISC1_ = 4,428 cell pairs, novel: *p* < 0.001, N_control_ = 1,697 cell pairs, N_tgDISC1_ = 4,292 cell pairs).
