# Supplementary material for: Disrupted‐in‐schizophrenia 1 overexpression disrupts hippocampal coding and oscillatory synchronization
Source: Hippocampus. 2019 Feb 5;29(9):802–16. doi: 10.1002/hipo.23076 (PMC6767395; doi:10.1002/hipo.23076)
Supplement: Supplementary file 1 — Supporting Information Figure S1 Behavior on the open‐field. (A) Movement speed (cm/sec) of control and tgDISC1 in the familiar and novel environments. In both environments tgDISC1 animals exhibited significantly lower speed (Wilcoxon Rank‐Sum test, Bonferroni‐Holm correction; familiar: p = 0.0002, novel: p = 0.01). (B) The percentage of immobility periods during familiar and novel environment exposure. Note that only periods where the animal moved more than 5 cm/s were included in the waking place field and oscillatory analyses. In both environments tgDISC1 rats spent significantly more time in immobility (Wilcoxon Rank‐Sum test, Bonferroni‐Holm correction; familiar: p = 0.012, novel: p = 0.022). (C) Percentage of the area of familiar and novel environments that were not visited by the animal. There were no differences in environment coverage between control and tgDISC1 animals (Wilcoxon Rank‐Sum test, familiar: p = 0.127, novel: p = 0.226). Supporting Information Figure S2: Novel environment exploration induces rate remapping. Cumulative probability distributions of the rate remapping scores for three different comparisons: familiar vs. novel (black), 1st vs. 2nd half of the familiar environment (blue) and 1st vs. 2nd half of the novel environment (red), for both control (left panel) and tgDISC1 (right panel) animals. Comparisons between the distributions are summarized in each panel (Two‐sample Kolmogorov–Smirnov test, P‐values are notated in the figure). Supporting Information Figure S3: TgDISC1 animals showed increased gamma power during familiar environment exploration as compared to controls. Normalized logarithmic power of the local field potential (0–500 Hz) for control (black) and tgDISC1 (green) animals during the exploration of familiar (top) and novel (bottom) environments is shown. Each line represents one recording session. The boxplots inside the panels denote the power distributions of the 1 Hz frequency bins comprising the theta (6–10 Hz) and gamma [file HIPO-29-802-s001.docx]

Disrupted-in-Schizophrenia 1 overexpression disrupts hippocampal coding and oscillatory synchronization

Kaefer and Malagon-Vina et al.

**Supporting Information**


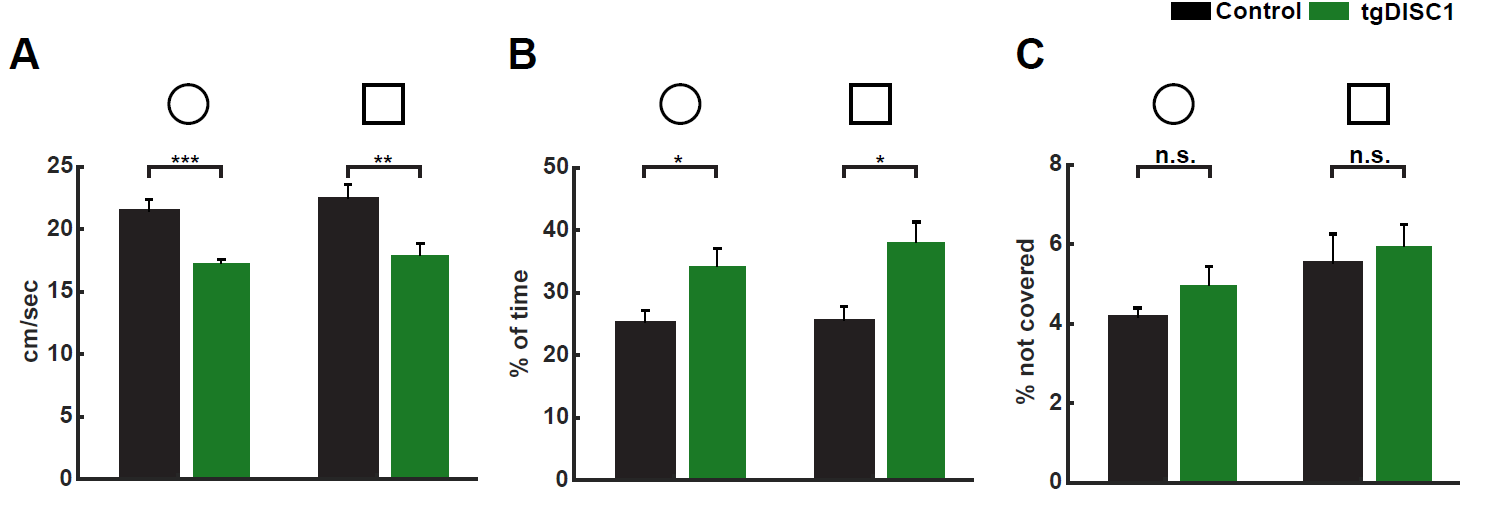


**Supporting Information Figure S1:** **Behavior on the open-field. (A)** Movement speed (cm/sec) of control and tgDISC1 in the familiar and novel environments. In both environments tgDISC1 animals exhibited significantly lower speed (Wilcoxon Rank-Sum test, Bonferroni-Holm correction; familiar: P = 0.0002, novel: P = 0.01). **(B)** The percentage of immobility periods during familiar and novel environment exposure. Note that only periods where the animal moved more than 5 cm/s were included in the waking place field and oscillatory analyses. In both environments tgDISC1 rats spent significantly more time in immobility (Wilcoxon Rank-Sum test, Bonferroni-Holm correction; familiar: P = 0.012, novel: P = 0.022). **(C)** Percentage of the area of familiar and novel environments that were not visited by the animal. There were no differences in environment coverage between control and tgDISC1 animals (Wilcoxon Rank-Sum test, familiar: P = 0.127, novel: P = 0.226).


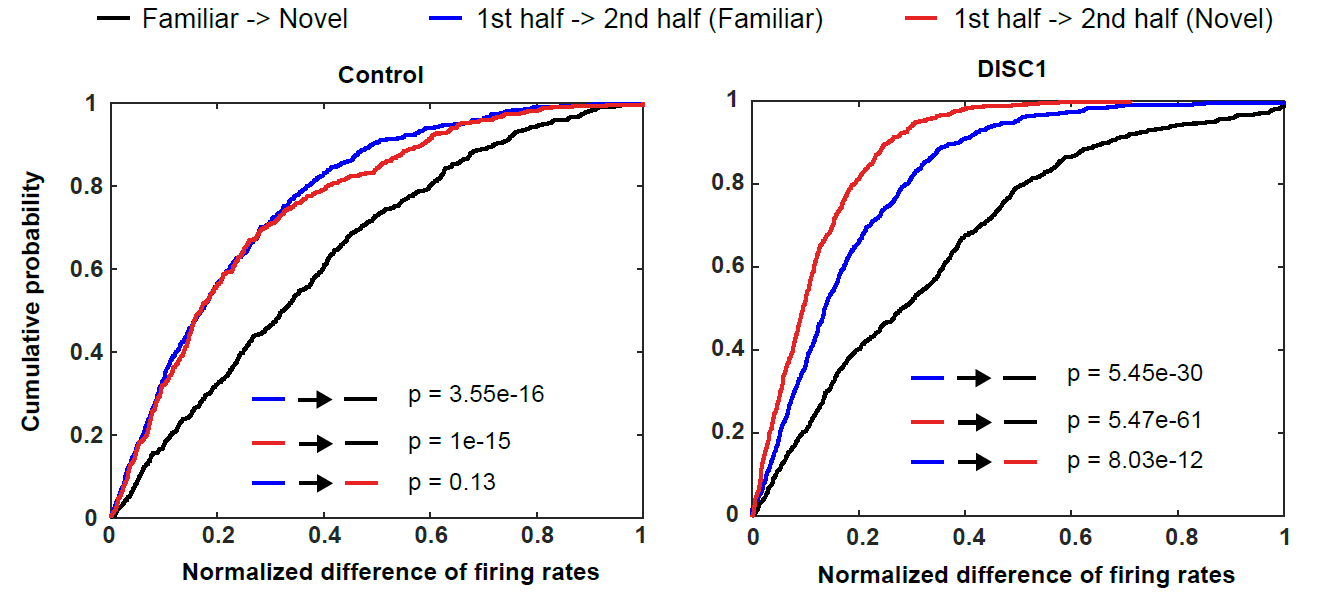


**Supporting Information Figure S2: Novel environment exploration induces rate remapping.** Cumulative probability distributions of the rate remapping scores for three different comparisons: familiar vs. novel (black), 1^st^ vs. 2^nd^ half of the familiar environment (blue) and 1^st^ vs. 2^nd^ half of the novel environment (red), for both control (left panel) and tgDISC1 (right panel) animals. Comparisons between the distributions are summarized in each panel (Two-sample Kolmogorov-Smirnov test, p-values are notated in the figure).


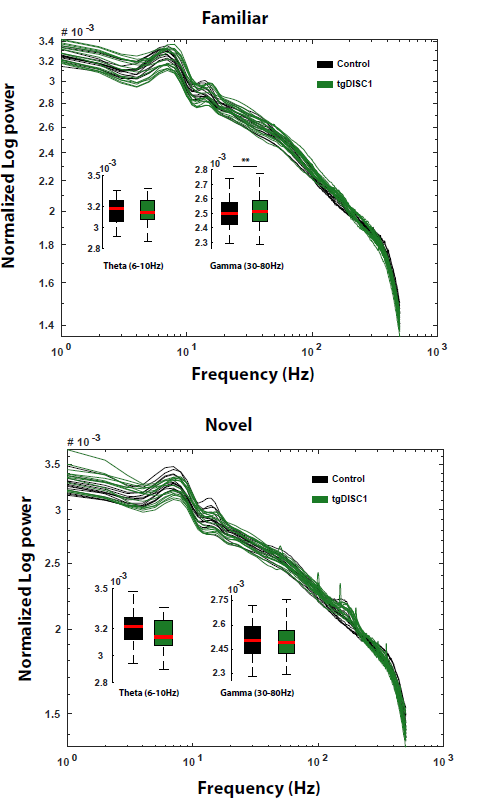


**Supporting Information Figure S3: TgDISC1 animals showed increased gamma power during familiar environment exploration as compared to controls.** Normalized logarithmic power of the local field potential (0 – 500 Hz) for control (black) and tgDISC1 (green) animals during the exploration of familiar (top) and novel (bottom) environments is shown. Each line represents one recording session. The boxplots inside the panels denote the power distributions of the 1 Hz frequency bins comprising the theta (6-10 Hz) and gamma (30-80 Hz) bands from all sessions. Stars indicate a significant difference between controls and tgDISC1 animals in the gamma band during the exploration of the familiar environment (Wilcoxon Rank-Sum test, Bonferroni-Holm correction; P = 0.0052).


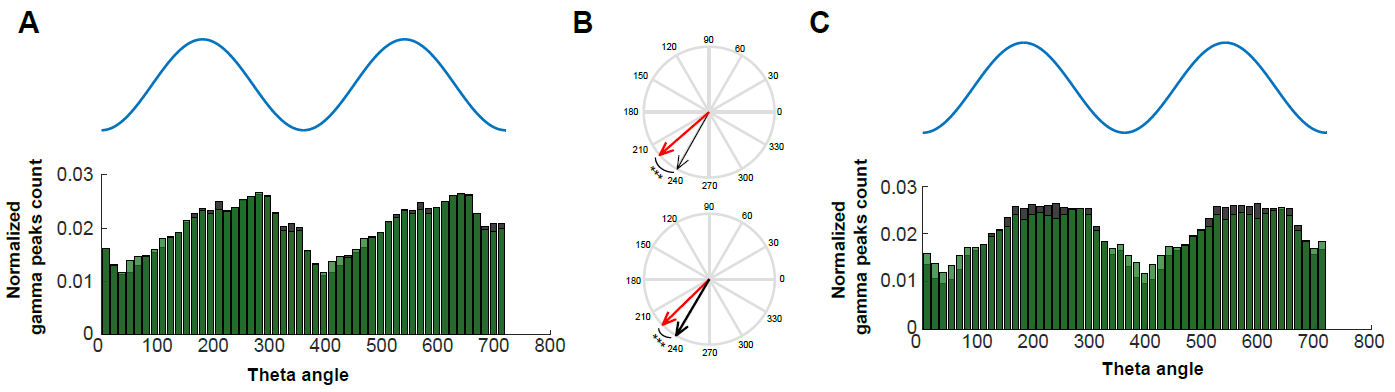


**Supporting Information Figure S4: Gamma oscillations lock differentially to theta in familiar and novel environments.** **(A)** Normalized histogram of the preferred theta phase (blue line represents two theta cycles) of gamma wave peaks, for control (black) and tgDISC1 (green) animals during familiar exploration. **(B)** Phase plots of the preferred theta locking phase of detected gamma wave peaks in familiar (black arrow) and novel (red arrow) environment, for controls (top) and tgDISC1 animals (bottom). Both groups of animals showed a significant shift of preferred theta locking phase of gamma waves (Watson-Williams test; controls: familiar (240°), novel (220°), P < 1e-30, tgDISC1: familiar (239°), novel (224°), P < 1e-30). **(C)** Same as **A,** but during novel environment exploration.


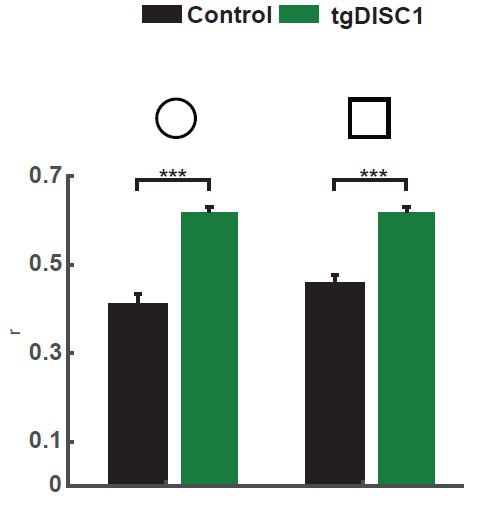


**Supporting Information Figure S5: Reactivation during rest period SWRs**. TgDISC1 animals showed significantly higher reactivation strength in sleep after both environments compared to controls (Z-test of Fisher z-transformed Pearson correlation coefficients, Bonferroni-Holm correction; familiar: P < 0.001, N_control_ = 1331 cell pairs, N_tgDISC1_ = 4428 cell pairs, novel: P < 0.001, N_control_ = 1697 cell pairs, N_tgDISC1_ = 4292 cell pairs).
